# The Role of Human Aldo-Keto Reductases in the Metabolic Activation and Detoxication of Polycyclic Aromatic Hydrocarbons: Interconversion of PAH Catechols and PAH *o-*Quinones

**DOI:** 10.3389/fphar.2012.00193

**Published:** 2012-11-16

**Authors:** Li Zhang, Yi Jin, Meng Huang, Trevor M. Penning

**Affiliations:** ^1^Center of Excellence in Environmental Toxicology, Department of Pharmacology, Perelman School of Medicine, University of PennsylvaniaPhiladelphia, PA, USA

**Keywords:** polycyclic aromatic hydrocarbons, *o-*quinones, aldo-keto reductases, conjugation reactions, phase II metabolism, redox-cycling

## Abstract

Polycyclic aromatic hydrocarbons (PAH) are ubiquitous environmental pollutants. They are procarcinogens requiring metabolic activation to elicit their deleterious effects. Aldo-keto reductases (AKR) catalyze the oxidation of proximate carcinogenic PAH *trans*-dihydrodiols to yield electrophilic and redox-active PAH *o-*quinones. AKRs are also found to be capable of reducing PAH *o-*quinones to form PAH catechols. The interconversion of *o-*quinones and catechols results in the redox-cycling of PAH *o-*quinones to give rise to the generation of reactive oxygen species and subsequent oxidative DNA damage. On the other hand, PAH catechols can be intercepted through phase II metabolism by which PAH *o-*quinones could be detoxified and eliminated. The aim of the present review is to summarize the role of human AKRs in the metabolic activation/detoxication of PAH and the relevance of phase II conjugation reactions to human lung carcinogenesis.

## Introduction

The Aldo-keto reductases (AKRs) are a superfamily of monomeric NAD(P)(H)-dependent oxidoreductases. They are cytosolic and have ∼320 amino acids with molecular weights at around 34–37 kDa (Jez et al., 1997). AKRs catalyze the reduction of aldehydes and ketones to yield primary and secondary alcohols on a variety of endogenous substrates and xenobiotics (Hara et al., 1996; Jin and Penning, 2007), and are formal phase I metabolic enzymes. AKRs have been implicated in a number of human diseases. AKR1B1 (aldose reductase) is implicated in the complications that arise due to diabetes, since it converts high blood glucose to the hyperosmotic sugar sorbitol (Lee et al., 1995; Suzen and Buyukbingol, 2003). AKR1B10 (retinal reductase) is involved in retinoic acid signaling and is implicated in the pathogenesis of lung cancer (Fukumoto et al., 2005; Penning and Lerman, 2008) and hepatocellular carcinoma (Liu et al., 2012). By contrast, AKR 1C family members play essential roles in metabolism of male and female sex hormones and may play roles in the development of hormone dependent malignancies of the prostate and breast (Penning and Byrns, 2009); while AKR1D1 (steroid 5β-reductase) is essential for bile-acid biosynthesis and inherited mutations in the *AKR1D1* gene are associated with bile-acid deficiency (Lemonde et al., 2003); The present review will focus on roles of AKRs in metabolism of polycylic aromatic hydrocarbons (PAH).

**Figure 1:**
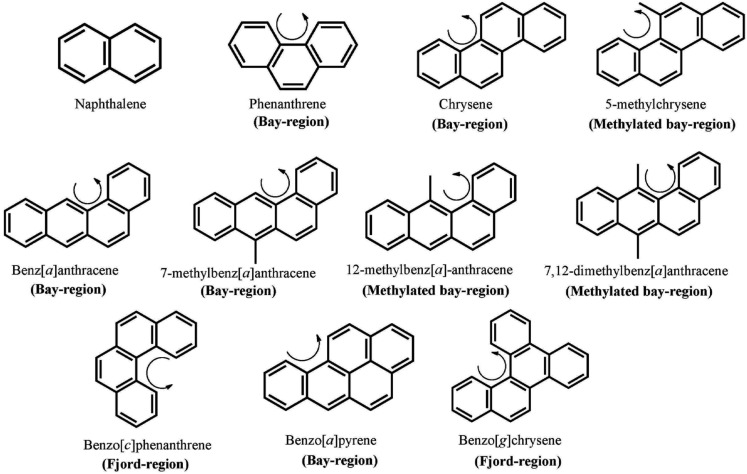
**Chemical structures of PAH**. The curly arrow denotes the presence of a bay-region; a methylated bay-region or a *fjord*-region.

**Figure 2:**
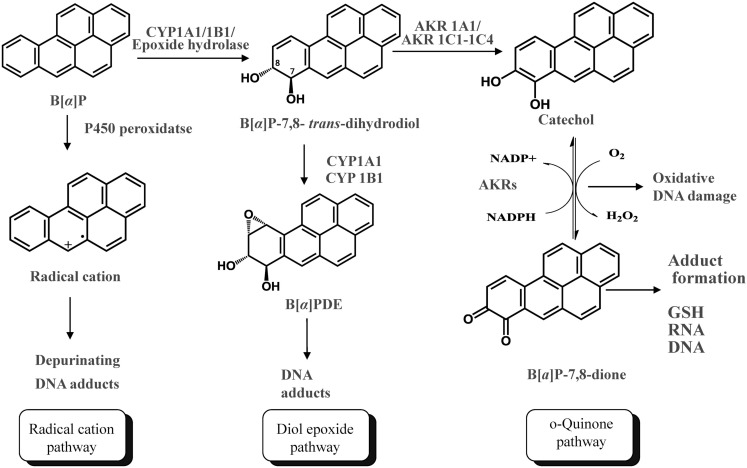
**Three Pathways of metabolic activation of PAH and interception by phase II enzymes**. (B[*a*]P is used as the representative PAH).

Polycylic aromatic hydrocarbons are ubiquitous environmental pollutants. They are suspect lung carcinogens and are products of tobacco smoke and incomplete fossil fuel combustion (Grimmer and Bohnke, 1975; Burczynski et al., 1999). PAH are characterized by the presence of two or more fused non-hetero aromatic rings arranged in various configurations (Fetzer, 2007). Based on the arrangement of their aromatic rings, PAH can be categorized into non-bay-region (e.g., naphthalene), bay-region (e.g., benzo[*a*]pyrene), and fjord-region (e.g., benzo[*g*]chrysene) PAH (Figure 1). Based on the number of the aromatic rings, the common PAH can be divided into the naphthalene (two rings), phenanthrene (three rings), chrysene, and 5-methyl-chrysene (four rings), benzo[*a*]pyrene (B[*a*]P), and benzo[*g*]chrysene (five rings) series, etc. Benzo[*a*]pyrene is a representative PAH and widely used to study the mutagenic and carcinogenic effects of PAH (Conney, 1982; Cavalieri and Rogan, 1995). PAH are not reactive and require metabolic activation to form electrophiles to elicit their deleterious effects, thus they are procarcinogens (Gelboin, 1980). There are three major pathways for the activation of B[*a*]P, which result in the formation of radical cations, diol epoxides, and electrophilic and redox-active *o-*quinones (Figure 2). In the present review, we will focus on the *o-*quinone pathway and discuss the role of human AKRs in the formation of the *o-*quinones, the redox-cycling of *o-*quinones to form catechols, and the removal of catechols by conjugating enzymes. For details about the other pathways, readers are referred to previous review papers (Penning et al., 1999; Penning, 2004; Xue and Warshawsky, 2005).

## Activation of PAH *trans*-Dihydrodiols by AKRs to form *o*-Quinones

In the *o*-quinone pathway of PAH activation, the proximate PAH carcinogens, *trans*-dihydrodiols, e.g., B[*a*]P-7,8-*trans*-dihydrodiol, are oxidized by AKRs to yield ketols which spontaneously rearrange to form catechols, e.g., B[*a*]P-7,8-catechol (Figure 2; Burczynski et al., 1998; Palackal et al., 2001, 2002). B[*a*]P-7,8-catechol is not stable and undergoes autoxidation to yield B[*a*]P-7,8-dione. PAH *o-*quinones are electrophilic and highly reactive to endogenous nucleophiles. PAH *o-*quinones can readily form conjugates with cellular thiols to yield l-cysteine, *N-*acetyl-l-cysteine (NAC), and GSH conjugates leading to their elimination (Murty and Penning, 1992a,b). PAH *o-*quinones can also react with DNA to form both stable and depurinating adducts *in vitro* which may result in mutagenesis (Shou et al., 1993; McCoull et al., 1999; Balu et al., 2006). PAH *o-*quinones are also able to undergo non-enzymatic/enzymatic reduction to reform catechols at the expense of NADPH and establish futile redox cycles which amplify the generation of reactive oxygen species (ROS). ROS can cause DNA damage resulting in the formation of 7,8-dihydro-8-oxo-2′-deoxyguanosine (8-oxo-dGuo) lesions, contributing to G-to-T transversions in *K-ras* and *p53* (Kasai et al., 1986; Cheng et al., 1992). PAH *o-*quinones were found to be more mutagenic than diol epoxides in an *in vitro* p53 mutagenesis assay and a linear correlation was observed between the mutagenic efficiency and the presence of 8-oxo-dGuo in the p53 cDNA (Yu et al., 2002; Park et al., 2006; Shen et al., 2006). More recently, the metabolic activation of B[*a*]P-7,8-*trans*-dihydrodiol to B[*a*]P-7,8-dione was demonstrated in human lung adenocarcinoma (A549) cells which shows high constitutive expression of AKRs. This metabolic activation led to the formation ROS and 8-oxo-dGuo lesions in cellular DNA (Park et al., 2008).

Several members of the AKR superfamily are able to oxidize PAH *trans*-dihydrodiols to *o-*quinones (Smithgall et al., 1986, 1988). The substrate specificity of AKRs covers structurally diverse PAH *trans*-dihydrodiols which range from the simplest *trans*-1,2-dihydroxy-1,2-dihydro-naphthalene, to bay-region dihydrodiols (e.g., *trans*-1,2-dihydroxy-1,2-dihydrochrysene), to methylated bay-region dihydrodiols (e.g., *trans*-3,4-dihydroxy-3,4-dihydro-7-methylbenz[*a*]anthracene), and to *fjord*-region dihydrodiols (e.g., *trans*-11,12-dihydroxy-11,12-dihydrobenzo[*g*]chrysene). In contrast, *K-*region dihydrodiols, in which the dihydroxy groups are located on a central benzo-ring, (e.g., *trans*-9,10-dihydroxy-9,10-dihydrophenanthrene and *trans*-4,5-dihydroxy-4,5-dihydroB[*a*]P) are not substrates of AKRs (Table 1; Palackal et al., 2001, 2002; Shultz et al., 2008).

**Table 1:** **Oxidation of PAH *trans*-dihydrodiols by human AKRs**.

PAH *trans*-dihydrodiols	ARK1A1	ARK1B1	ARK1B10	ARK1C1	ARK1C2	ARK1C3	ARK1C4	AKR7A2	AKR7A3
	
	k_cat_/*K*_m_ (mM^−1^ min^−1^)								
Naphthalene-1,2-diol	10.4^a^	NA	NA	151^b^	100^b^	6.4^b^	32.7^b^	NA	NA
**NON-*K*-REGION DIHYDRODIOLS**									
Phenanthrene-1,2-diol	17.9^a^	NA	NA	ND^b^				NA	NA
Chrysene-1,2-diol	15.5^a^	NA	NA	ND^b^	7.03^b^	4.26^b^	10^b^	NA	NA
Benz[*a*]anthracene-3,4-diol	(−) 68.1^a^	NA	(+) 12.8^d^	9.5^b^	17.6^b^	18.0^b^	32.2^b^	NA	NA
Benzo[*a*]pyrene-7,8-diol	(−) 29.6^a^	(+) 10.3^d^	(+) 2.36^d^	22.6^b^	53.3^b^	24.7^b^	16.7^b^	ND^c^	ND^c^
**METHYLATED DERIVATIVES**									
7-Methylbenz[*a*]anthracene-3,4-diol	(+) 85.8^a^	NA	NA	4.8^b^	49.5^b^	30.1^b^	46.9^b^	NA	NA
12-Methylbenz[*a*]-anthracene-3,4-diol	ND^a^	NA	NA	ND				NA	NA
7,12-Dimethylbenz[*a*]anthracene-3,4-diol	(−) 97.1^a^	NA	2.7^d^	7.4^b^	46.8^b^	19.7^b^	185^b^	NA	NA
5-Methylchrysene-7,8-diol	130^a^	NA	NA	12.4^b^	28.8^b^	9.0^b^	35.2^b^	NA	NA
***K*-REGION DIHYDRODIOLS**									
Phenanthrene-9,10-diol	ND^a^	NA	NA	ND^b^				NA	NA
Benzo[*a*]pyrene-4,5-diol	ND^a^	NA	NA	ND^b^				NA	NA
**FJORD-REGION DIHYDRODIOLS**									
Benzo[*c*]phenanthrene-3,4-diol	11.8^a^	NA	1.5^d^	ND^b^	4.3^b^	6.6^b^	8.2^b^	NA	NA
Benzo[*g*]chrysene-11,12-diol	11.3^a^	NA	9.55^d^	4.5^b^	4.9^b^	23^b^	165^b^	NA	NA

*^a^Palackal et al., 2001, ^b^^2002^, ^c^Shultz et al., 2011, ^d^Quinn et al., 2008; ND: not detected; + or – in parenthesis, stereospecificity of AKR to the PAH *trans*-dihydrodiols where no parenthesis exist the AKR isoform oxidizes both isomers of the racemic mixture*.

In considering the human enzymes, AKR1A1 was stereoselective and will only oxidize (−)-B[*a*]P-7(*R*),8(*R*)-dihydrodiol, which is the major stereoisomer formed *in vivo*. Similarly, AKR1A1 oxidized (−)-benz[*a*]anthracene-3(*R*),4(*R*)-dihydrodiol, (+)-7-methylbenz[*a*]anthracene-3(*S*),4(*S*)-dihydrodiol, and (−)-7,12-dimethylbenz[*a*]anthracene-3(*R*),4(*R*)-dihydrodiol rather than both diastereomers (Table 1; Palackal et al., 2001).

AKR1B10 is one of the most overexpressed genes in non-small lung carcinoma and a member of the smoking gene battery that is up-regulated in response to cigarette smoking and down-regulated in smokers who quit (Fukumoto et al., 2005; Zhang et al., 2008). AKR1B10 was found to oxidize a wide range of PAH *trans*-dihydrodiol substrates *in vitro* to yield PAH *o-*quinones, but showed improper stereospecificity with B[*a*]P-7,8-dihydrodiol in that it only oxidized the minor (+)-B[*a*]P-7(*S*),8(*S*)-dihydrodiol isomer. The related subfamily member AKR1B1 displayed the same stereochemical specificity as AKR1B10 on racemic B[*a*]P-7,8-*trans*-dihydrodiols (Quinn et al., 2008). The stereochemical preference of AKR1B10 appears to be limited only to B[*a*]P-7,8-*trans*-dihydrodiol and benzo[*a*]anthracene-3,4-diol, since no stereospecificity for the oxidation of the (−)-*R,R* and (+)-*S,S* stereoisomers of benzo[*g*]chrysene-11,12-dihydrodiol and 7,12-dimethylbenz[*a*]anthracene-3,4-diol was noted.

Four human AKR1C subfamily members (AKR1C1-AKR1C4) oxidized B[*a*]P-7,8-*trans*-dihydrodiol to B[*a*]P-7,8-dione in the following rank order: AKR1C2 > AKR1C1 ∼ AKR1C3 > AKR1C4 (Burczynski et al., 1998). AKR1C1-AKR1C4 oxidized both stereoisomers of racemic *trans*-dihydrodiols, although AKR1C1 and AKR1C2 displayed a preference for the (+)-B[*a*]P-7(*S*),8(*S*)-dihydrodiol isomer (Burczynski et al., 1998). AKR1C1-AKR1C4 showed high activity for both stereoisomers of the bay-region substituted PAH *trans*-dihydrosiols, where bay-region substituted PAH are more carcinogenic than B[*a*]P (Table 1; Palackal et al., 2002).

## Reduction of PAH *o*-Quinones by AKRs

The metabolic activation of B[*a*]P-7,8-*trans*-dihydrodiol to B[*a*]P-7,8-dione was demonstrated in human lung adenocarcinoma A549 cells which show high constitutive expression of AKRs (Park et al., 2008). This metabolic activation led to the formation of ROS and 8-oxo-dGuo lesions in cellular DNA. Importantly, oxidative stress was exacerbated in the presence of a catechol-*O-*methyl transferase (COMT) inhibitor (Park et al., 2008). This observation indicated that the redox-cycling between B[*a*]P-7,8-dione and B[*a*]P-7,8-catechol occurred with a concomitant generation of ROS which in turn resulted in DNA damage. COMT was able to intercept the catechol and thus protect against the insult from redox-cycling. When B[*a*]P-7,8-dione was given to human bronchoalveolar H358 cells, similar exacerbation of cellular oxidative DNA damage was observed in the presence of a COMT inhibitor (Mangal et al., 2009). Both COMT cell-based studies clearly suggest that two electron reduction of the PAH *o-*quinone to the PAH catechol not only results in oxidative stress and DNA damage, but also leads to *O*-methylation and detoxication of PAH *o-*quinones at the level of PAH catechols (Figure 2).

The enzymatic two electron reduction of quinones to hydroquinones is thought to be able to protect against quinone-induced cellular oxidative stress, because the hydroquinone would be available for phase II conjugation reactions. However, if the rates of conjugation reactions are overwhelmed by the rate of the ensuing redox-cycling, the reduction process may be deleterious (Figure 2). It is not well understood which enzymes account for the process of two electron reduction of PAH *o-*quinones to PAH catechols and contribute to redox-cycling. Candidate enzymes that may catalyze this reduction include NAD(P)(H):quinone oxidoreductase (NQO1), carbonyl reductases (CBR1 and CBR3), and AKRs. In order to identify the enzymes responsible for the reduction of the PAH *o-*quinones, the ability of homogeneous recombinant NQO1, CBRs, and AKRs to reduce PAH *o-*quinones were compared (Shultz et al., 2011). Except for discrete *o-*quinones, the rank order of activity was: NQO1 > AKR7A2 > CBRs.

NQO1 is a flavoenzyme that catalyzes two electron reduction of quinones to hydroquinones by using NAD(P)H as an electron donor (Jaiswal et al., 1988). Despite its high *o-*quinone reductase activity, NQO1 did not appear to be the dominant enzyme that catalyzes *o-*quinone reduction in human lung A549 cells since treatment with NQO1 inhibitor, dicumarol did not eliminate the deleterious ROS generated by PAH *o-*quinone redox-cycling (Shultz et al., 2011) suggesting that AKRs and CBRs could be the culprit enzymes.

CBRs are cytosolic, monomeric oxidoreductases that catalyze the reduction of a large number of carbonyl compounds (Wermuth, 1981). Human placental CBR1 (15-hydroxyprostaglandin dehydrogenase/prostaglandin 9-ketoreductase) catalyzed the reduction of the non-*K*-region *o-*quinone such as B[*a*]P-7,8-dione (Jarabak, 1991, 1992). However, studies using purified human recombinant CBR1 showed that the substrate specificity of CBR was quite narrow and it reduced *K*-region *o-*quinones but not the non-*K*-region *o-*quinones which are products of PAH *trans*-dihydrodiol oxidation catalyzed by AKRs (Shultz et al., 2011). This suggests that CBR would not play a critical role in the two electron reduction of PAH *o-*quinones.

AKR1C9 (rat liver 3α-hydroxysteroid/dihydrodiol dehydrogenase) was first found to catalyze the reduction of B[*a*]P-7,8-dione at an unexpectedly staggering rate of 4750 nmol/min/mg which was three orders of magnitude greater than the rate of conversion of B[*a*]P-7,8-*trans*-dihydrodiol to B[*a*]P-7,8-dione catalyzed by the same enzyme (Smithgall et al., 1986; Flowers-Geary et al., 1992). In further studies, a panel of purified human AKRs (AKR 1A1, 1B1, 1B10, 1C1-1C4, 7A2, and 7A3) were shown to catalyze the reduction of B[*a*]P-7,8-dione and other PAH *o-*quinones with the specific activities that were 100–1000 times greater than their respective activities to oxidize the cognate PAH *trans*-dihydrodiol (Shultz et al., 2011). Of all AKRs studied, AKR7A2 is the most efficient enzyme for the reduction of B[*a*]P-7,8-dione (Table 2).

**Table 2:** **Reduction of B[*a*]P-7,8-dione by human AKRs**.

AKR	B[*a*]P-7,8-dione reduction (nmol/min/mg)
AKR1A1	350
AKR1B1	250
AKR1B10	250
AKR1C1	64
AKR1C2	350
AKR1C3	130
AKR1C4	130
AKR1D1	ND
AKR7A2	1270
AKR7A3	1170

*Data from: Shultz et al., 2011*.

The AKRs exhibited different reductase activities on series of PAH *o-*quinones which included phenanthrene, chrysene, pyrene, and anthracene series (Shultz et al., 2011). By comparing the ability of AKRs to reduce B[*a*]P-7,8-dione and their ability to oxidize B[*a*]P-7,8-*trans*-dihydrodiol, it was noted that the AKR with the highest quinone reductase activity on a particular PAH *o-*quinone was not always identical to the AKR isoform with the highest dihydrodiol dehydrogenase activity for the respective PAH-*trans*-dihydrodiol. For example, AKR7A2 and AKR7A3 exhibited the highest specific activities for B[*a*]P-7,8-dione reduction, but failed to catalyze the oxidation of PAH-*trans*-dihydrodiols (Shultz et al., 2011; Tables 1 and 2). The two electron reduction of PAH *o-*quinones catalyzed by AKRs was demonstrated to lead to futile redox cycles. In each instance, 10 μM PAH *o-*quinone consumed 180 μM NADPH, and the consumption of cofactor was accompanied by a concomitant consumption of molecular oxygen and the production of superoxide anion and hydrogen peroxide (Shultz et al., 2011).

The contribution of individual AKRs to the redox-cycling, if PAH *o-*quinones are in lung, will depend on their levels of expression and catalytic efficiency for each PAH *o-*quinone substrate. Although AKR1A1 catalyzes the most effective oxidation of the major enantiomer of B[*a*]P -*trans*-dihydrodiol *in vivo*, (−)-B[*a*]P-7(*R*),8(*R*)-dihydrodiol (Palackal et al., 2001), it has the lowest quinone reductase activity among all AKRs for most PAH *o-*quinones (Shultz et al., 2011). Also, the expression level of AKR1A1 in normal human bronchoalveolar cells is very low (Jiang et al., 2006; Quinn and Penning, 2008), which implies that AKR1A1 is not critical in the enzymatic reduction of PAH *o-*quinones in the lung.

AKR1B10 has a wide substrate specificity for PAH *o-*quinones and exhibits high catalytic efficiency for PAH *o-*quinones particularly for the chrysene series (Shultz et al., 2011). As it is up-regulated in response to tobacco smoke exposure (Fukumoto et al., 2005; Gumus et al., 2008; Zhang et al., 2008), AKR1B10 may play an important role in ROS generation from PAH *o-*quinone redox cycling in lung cells. However, AKR1B1 and AKR1B10 only oxidize the minor isomer (+)-B[*a*]P-7(*S*),8(*S*)-dihydrodiol formed *in vivo* with low catalytic efficiency (Quinn et al., 2008), suggesting that AKR1Bs are not as important in the oxidation of B[*a*]P *trans*-dihydrodiols as other AKRs.

Among all AKRs, AKR1C1-1C3 are generally the most efficient isoforms to catalyze the oxidation of PAH *trans*-dihydrodiols (Palackal et al., 2002). They are able to convert both isomers of racemic PAH *trans*-dihydrodiols formed *in vivo* to *o-*quinones (Palackal et al., 2001). AKR1C1-1C3 also display medium to high specific activities for the reduction of most PAH *o-*quinones tested excluding the anthracene series and dibenzo[*a,c*]-phenanthrene-3,4-dione (Shultz et al., 2011). It was found that the expression levels of AKR1C1-1C3 in A549 cells, though lower than that of AKR1B10, were significant higher than AKR1A1 and AKR7A2, suggesting AKR1C isoforms may also be important in catalyzing redox-cycling of PAH *o-*quinones in the lung (Quinn et al., 2008).

Although the expression of AKR7A2 is low in A549 cells, its superior catalytic efficiency for most of PAH *o-*quinones as well as its capability of reducing dimethylbenz[*a*]anthracene-3,4-dione and benz[*a*]anthracene-3,4-dione which are non-substrates of other AKRs may make it play a role in the reduction of these PAH *o-*quinones in lung (Quinn et al., 2008; Shultz et al., 2011).

## Detoxication of PAH *o*-Quinones by Human COMT

The observation that ROS generation from PAH *o-*quinone in A549 cells was exacerbated by a COMT inhibitor infers that metastable PAH catechols are formed in lung cells and that these catechols can be intercepted by COMT (Park et al., 2008). COMT is a classical phase II enzyme and catalyzes the transfer of a methyl group from *S*-adenosyl-l-methionine (SAM) to the hydroxyl group of a variety of catechols including catecholamine neurotransmitters and the catechol estrogens (Axelrod and Tomchick, 1958; Axelrod, 1966; Ball et al., 1972). There are two major COMT isoforms in human, the soluble cytosolic form (S-COMT), and the membrane-bound endoplasmic reticulum form (MB-COMT), encoded by a single gene at 22q11.2 (Grossman et al., 1992; Tenhunen et al., 1994). The two isoforms share identical amino acid sequences except that the MB-COMT contains an NH_2_ terminal extension of 50 amino acids to serve as a hydrophobic anchor to the membrane (Ulmanen and Lundstrom, 1991). COMT is widely distributed among various organs in the body including lung where high COMT activity was found (Mannisto and Kaakkola, 1999). Except in brain, S-COMT is the predominant form in most tissues (Jeffery and Roth, 1984; Grossman et al., 1985; Tenhunen and Ulmanen, 1993).

Polycylic aromatic hydrocarbons *o-*quinones are structurally related to the estrogen *o-*quinones, which demonstrate similar genotoxic modes of action (Penning et al., 1999; Bolton et al., 2000; Bolton and Thatcher, 2008). The formation and detoxication of estrogen *o-*quinones are well studied and can be used as parallel for the studies of PAH *o-*quinone detoxication. The biotransformation of estrogens such as 17β-estradiol and estrone is primarily catalyzed via P450 pathways to yield the 2-hydroxy and 4-hydroxyl catechol estrogens (Aoyama et al., 1990; Kerlan et al., 1992; Martucci and Fishman, 1993; Shou et al., 1997). Both catechol estrogens can be further oxidized to an estrogen *o-*quinone which could form stable and depurinating DNA adducts (Liehr et al., 1986; Stack et al., 1996; Cavalieri et al., 1997). The detoxication of catechol estrogens can occur by *O-*methylation catalyzed by COMT (Schneider et al., 1984; Dawling et al., 2001).

**Figure 3:**
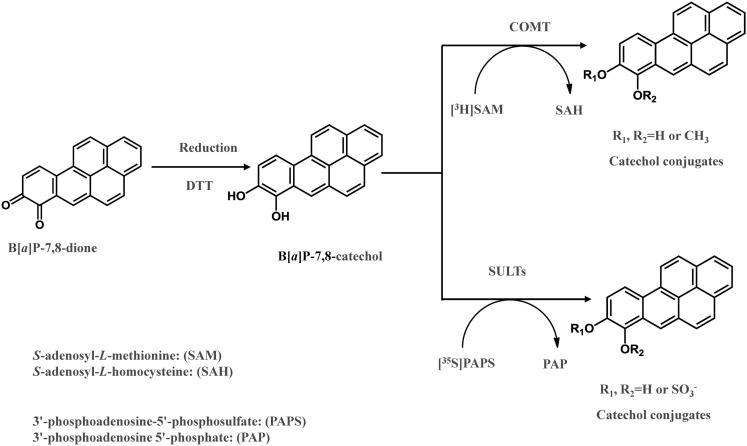
***In vitro* COMT and SULT assay to measure *O-*methylation and *O-*sulfation of PAH *o*-quinones**.

The detoxication of PAH *o-*quinines by COMT was investigated recently (Zhang et al., 2011). B[*a*]P-7,8-dione was reduced to the catechol by dithiothreitol anaerobically in the presence of *S*-adenosyl-l-methionine and further *O-*methylated by human recombinant COMT (Zhang et al., 2011; Figure 3). COMT showed quite a wide substrate specificity and *O-*methylated a series of structurally diverse PAH catechols such as bay-region, methylated bay-region and fjord-region PAH catechols. PAH catechols often formed two isomeric products. For B[*a*]P-7,8-catechols, the two products were formed at a ratio of 9:1 with the major metabolite being *O-*8-monomethyl-B[*a*]P-7,8-catechol (Zhang et al., 2011).

Catalytic efficiencies (*k*_cat_/*K*_m_) of *O-*methylation of PAH catechols by COMT varied greatly among the classes of PAH catechols studied (Table 3). PAH catechols containing a methylated bay-region or a *fjord*-region, which have bent structures due to steric clashing of bay-region hydrogen atoms, have high efficiency of *O*-methylation. However, pronounced substrate inhibition was also observed with these PAH catechols. Since substrate inhibition occurs at low micromolar concentrations, these PAH catechols may not be efficiently detoxified by COMT and thus are more likely to undergo redox-cycling to cause ROS generation (Zhang et al., 2011).

**Table 3:** ***O*-methylation of PAH catechols by COMT**.

Quinone	*k*_cat_/*K*_m_	Substrate inhibition	M1^a^	M2^b^
	(min^−1^ μM^−1^)		(%)	(%)
Naphthalene-1,2-dione	4.9	−	100	0
Chrysene-1,2-dione	1.7	+	62	38
Chrysene-3,4-dione	0.02	−	83	16
5-Methyl-chrysene-7,8-dione	10.1	+	55	45
Benz[*a*]anthracene-3,4-dione	4.0	+	59	41
7-Methylbenz[*a*]anthracene-3,4-dione	1.6	+	53	47
12-Methylbenz[*a*]anthracene-3,4-dione	9.0	+	62	38
7,12-Dimethylbenz[*a*]anthracene-3,4-dione	6.8	+	32	68
Benzo[*c*]phenanthrene-3,4-dione	3.5	−	67	34
B[*a*]P-7,8-dione	0.7	−	90	10
Benzo[*g*]chrsyene-11,12-dione	8.0	+	36	64
Pyrocatechol	0.2	−	NA	NA

*a%, Product as isomer 1; b%, Product as isomer 2*.

*+, Where substrate inhibition is observed; −, substrate inhibition is not observed*.

*Data from: Zhang et al., 2011*.

The human *COMT* gene has a common G to A polymorphism that results in valine to methionine substitution at residue 108 for S-COMT or residue 158 for MB-COMT. Compared with the wild type, the Met/Met homozygous COMT activity in red blood cells was reduced by half, and the Met/Val heterozygous COMT showed intermediate activity for 3,4-dihydroxybenzoic acid (Syvanen et al., 1997). The low activity of the COMT mutant is related to its poor thermostability at physiological temperature, and not due to different kinetic properties. This SNP in the *COMT* gene has been associated with an increased risk of lung cancer (Zienolddiny et al., 2008; Cote et al., 2009). As COMT can act as a detoxication enzyme for PAH catechols, it is possible that these polymorphic variants may increase susceptibility to lung cancer caused by PAH.

## Detoxication of PAH *o*-Quinones by Human Sulfotransferases and Uridine Diphosphate Glucuronosyltransferases

While methylation of estrogen catechols has been found as an important pathway for detoxication of estrogen *o-*quinones, both sulfate and glucuronide conjugates of estrogen catechols catalyzed by the human sulfotransferases (SULTs) and uridine diphosphate glucuronosyltransferases (UGTs), respectively, has been observed (Brueggemeier et al., 1984; Adjei and Weinshilboum, 2002; Taskinen et al., 2003; Hui et al., 2008). Since PAH *o-*quinones are structurally related to the estrogen *o-*quinones, it is very likely that sulfation and glucuronidation of PAH catechols represent other pathways of detoxication of PAH *o-*quinones. SULTs are a group of cytosolic enzymes responsible for the transfer of a sulfonate group from 3′-phosphoadenosine 5′-phosphosulfate (PAPS) to either a hydroxyl moiety or an amine group (Negishi et al., 2001). SULTs catalyze the sulfate conjugation of steroid hormones, neurotransmitters, drugs, and xenobiotic compounds (Coughtrie et al., 1998). On the basis of amino acid sequence identity, human SULTs are divided into two main families SULT1 and SULT2, which are also termed the phenol sulfotransferase and the hydroxysteroid sulfotransferase family, respectively. SULT enzymes have a broad tissue distribution including liver, lung, brain, skin, etc. (Cappiello et al., 1989).

In a recent study, it was revealed that SULT1A1, 1A3, and 1E1, were expressed in human lung adenocarcinoma A549 cells, human bronchoalveolar H358 cells, immortalized human bronchial epithelial cells (HBEC-KT), and normal human bronchial epithelial cells (BEAS-2B; Zhang et al., 2012). When B[*a*]P-7,8-dione was reduced anaerobically to the catechol, it was found to be a substrate for these three human recombinant SULTs, and produced two *O*-sulfated products (Zhang et al., 2012). In these assays, the metastable PAH catechol was generated anaerobically and further sulfated by SULTs using PAPS as the sulfate donor (Figure 3). Two isomeric mono-*O-*sulfated-B[*a*]P-7,8-catechols were generated and their identities were confirmed by LC-MS-MS and 2D[^1^H]NMR. The major metabolite formed by SULT1A3 was found to be 8-hydroxy-B[*a*]P-7-*O-*sulfate with the minor metabolite being 7-hydroxy-B[*a*]P-8-*O-*sulfate. SULT1A1 only generated the 8-hydroxy-B[*a*]P-7-*O-*sulfate metabolite. SULT1E1 generated similar amounts of both isomers. SULTs displayed *K*_m_ values in the low micromolar or sub-micromolar range which were compatible or even lower than those for estrogen catechols (Zhang et al., 2012). The studies indicate that sulfation of PAH catechols by SULTs could be an important phase II pathway for the detoxication of PAH *o-*quinones, and that the major enzyme involved was SULT1A1.

SULT1A1 polymorphism has been associated with increased lung cancer risk (Wang et al., 2002). The common SULT1A1 allozymes consist of *1 (wild type), *2 variant (Arg213His), and *3 variant (Met223Val; Carlini et al., 2001). The allelic frequencies for SULT1A1*1,*2,*3 in Caucasian were 0.656, 0.332, 0.012, respectively. Despite the low frequency of SULT1A1*3 in Caucasians, it has an allelic frequency of 0.229 in African-Americans (Carlini et al., 2001). It has been reported that SULT1A1 recombinant allozymes have variable thermal stability and specific activity toward *p*-nitrophenol, catechol estrogens, and dietary flavonoids (Raftogianis et al., 1999; Adjei and Weinshilboum, 2002; Nagar et al., 2006). The SULT1A1*2 variant was associated with low enzyme activity and thermal stability (Raftogianis et al., 1999; Wang et al., 2002; Nagar et al., 2006). Although SULT1A1*3 had compatible thermal stability of the wild type, its specific activities for SULT1A1 substrates were lower than that of the wild type in many cases (Nagar et al., 2006). Our study showed that the catalytic efficiency of SULT1A1*3 with B[*a*]P-7,8-catechol was about 50% of the wild type SULT1A1. Therefore, polymorphic variants of SULT1A1 may have reduced efficiency to detoxify PAH *o-*quinones. Unlike the high allelic frequencies of SULT1A1 variants, SULT1A3, and SULT1E1 variants were found to be very rare which suggests that genetic polymorphism of these two enzymes may have minimal effect on PAH *o-*quinone detoxication (Glatt and Meinl, 2004; Hildebrandt et al., 2004).

Uridine diphosphate glucuronosyltransferases are superfamily of microsomal enzymes catalyzing the glucuronidation of a variety of endogenous compounds and xenobiotics (King et al., 2000). Based on sequence identities, UGTs are divided into two main subfamilies, UGT1 and UGT2 (Tukey and Strassburg, 2000). UGTs are widely distributed in a variety of tissues, including liver, intestine brain, kidney, lung, etc. (Guillemette, 2003). Several UGTs were found to catalyze the glucuronidation of PAH mono-phenols and dihydrodiols (Zheng et al., 2001; Olson et al., 2011). The major enzyme isoforms that glucuronidate PAH catechols remain to be identified.

## Detoxication of PAH *o-*Quinones in Human Lung Cells

The existence of Phase II detoxication of PAH *o-*quinones was confirmed in human lung cells (A549, H358, and HBEC-KT cells; Huang et al., 2012b). Consistent with the studies that used human recombinant enzymes, both mono-8-*O-*methylated B[*a*]P-7,8-catechol and mono-8-hydroxy-B[*a*]P-7-*O-*sulfate were formed in three human lung cells. The detection of these metabolites in human lung cells suggests that *O-*methylation and sulfation of PAH catechols are critical pathways in detoxication of PAH *o-*quinones in human lung. Evidence for the formation PAH catechol glucuronides was also found, although absolute chemical structures of them require elucidation.

**Figure 4:**
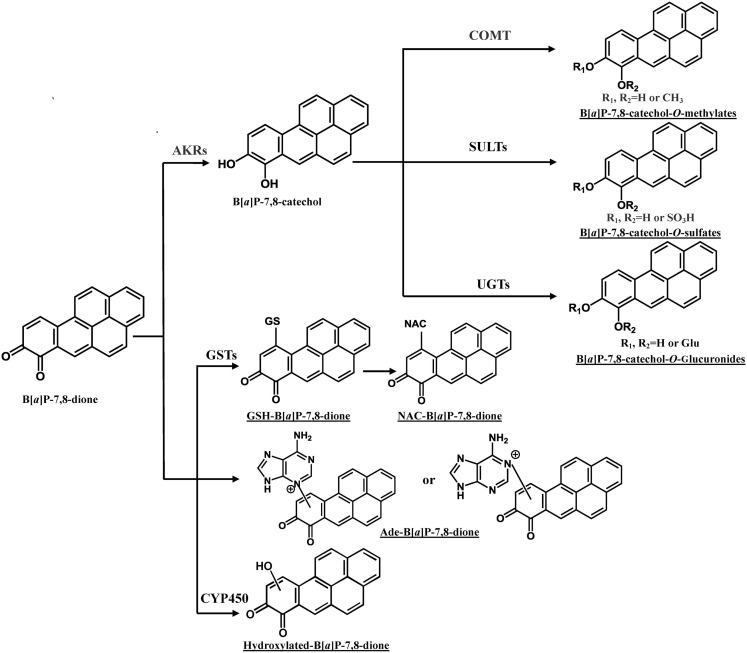
**Metabolic pathways of B[*a*]P-7,8-dione in human lung cells**. Metabolites of B[*a*]P-7,8-dione detected from human lung cells are underlined (Huang et al., 2012b). Ade, adenosine.

In addition to the formation of *O-*methylated, *O-*sulfate, and *O-*glucuronide conjugates of B[*a*]P-7,8-catechol, the glutathione (GSH) conjugate, NAC conjugate, and a B[*a*]P-7,8-dione adenine adduct were also detected in human lung cells (Figure 4; Huang et al., 2012a,b). PAH *o-*quinones were reported to readily form thioether conjugates with l-cysteine and GSH conjugates *in vitro* (Smithgall et al., 1986; Murty and Penning, 1992a,b). Thio-conjugation occurred at C10 of B[*a*]P-7,8-dione (Murty and Penning, 1992a). The GSH and NAC conjugates of B[*a*]P-7,8-dione formed in the human lung cells were found to be identical to those obtained from non-enzymatic synthesis (Huang et al., 2012b). However, glutathione *S*-transferase (GSTs) may also be involved. To form the NAC conjugate of B[*a*]P-7,8-dione, the GSH conjugate would be converted into a Cys-Gly conjugate by γ-glutamyltranspeptidase, and then further metabolized into a Cys conjugate by the action of a dipeptidase, and ultimately the NAC conjugate would be formed by *N*-acetyl transferase (Blair, 2006, 2010). Future studies will be required to identify the GST isoforms involved in the thio-conjugation of PAH *o-*quinones. Although thio-conjugation of *o-*quinones could enhance the polarity and solubility of PAH *o-*quinones to facilitate the disposition of PAH, the ability of these *o-*quinone thioether conjugates to redox-cycling remains (Monks and Lau, 1997). It was shown that GSH conjugates of benzoquinone undergo redox-cycling to produce renal toxicity. In this respect, thioether conjugates are not completely innocuous. 1,4-Michael addition of PAH *o-*quinones with DNA could also give rise to depurinating and stable DNA adducts. Treatment of lung cells (A549, H358, and HBEC-KT) with 2 μM B[*a*]P-7,8-dione consistently generated a B[*a*]P-7,8-dione adenine adduct (Huang et al., 2012a). Sources of this adduct other than DNA exist, since adenine is a key component of NAD(P)(H) and ATP, and the acid conditions used in its isolation could lead to glycosidic and ester bond cleavage. Thus it is not possible to conclude that this adduct came from DNA.

Our data support the concept that AKRs not only activate PAH *trans*-dihydrodiols by forming redox-active PAH *o-*quinones, but also facilitate the redox-cycling of the PAH *o*-quinones to catechols. The catechols are available for conjugation by a wide range of Phase II enzymes (Figure 2). Phase II conjugation of PAH catechols will significantly alter the detrimental properties of PAH *o-*quinone on lung cells. First, it terminates the futile redox-cycling of PAH *o-*quinones that leads to ROS generation and subsequent oxidative DNA damage. Second, it eliminates the electrophilicity of PAH *o-*quinone and prevents the formation of covalent adducts with protein and DNA. Finally, glucuronidation and sulfation usually result in more polar metabolites with enhanced renal or biliary excretion of xenobiotics or drugs, thus conjugation of PAH catechols may also facilitate elimination of PAH *o-*quinones from the body. Since AKRs are involved in activation and deactivation of PAH, it is important to study the expression of AKRs in human lung cells so as to understand the contribution of each AKR isoform in toxification and detoxication of PAH in lung. Except liver-specific AKR1C4, AKR1C1, 1C2, 1C3 were found to be highly expressed in human lung tissue (Penning et al., 2000) were overexpressed in non-small-cell lung carcinoma (Fukumoto et al., 2005; Woenckhaus et al., 2006), and can be induced by PAH (Burczynski et al., 1999; Courter et al., 2007; Misaki et al., 2007; Machala et al., 2008). Transcript levels of AKR1A1, AKR1C, AKR1B, and AKR7A2 isoforms were compared in A549 cells (Quinn et al., 2008). AKR1B1 and particularly AKR1B10 were the most abundant AKR isoforms followed by AKR1C isoforms, while the expression of AKR1A1 and 7A2 were much lower than AKR1B and 1C isoform expression. A549 cells were also found to have significantly greater AKR1B10 transcript levels than found in HBEC-KT cells which are more similar to normal lung epithelium cells. As human lung adenocarcinoma cells, A549 cells may not accurately reflect enzyme levels of normal human lung, further studies are required to investigate expression of the AKRs in cell models that better represent normal human lung cells.

Animal models such as rats and mice are often used to study PAH carcinogenesis raising the possibility that more could be learned by the use of AKR knockout or AKR transgenic mice. However, caution should be exercised when rodent models are used to draw conclusions as to the role of human AKRs in PAH carcinogenesis. The most important enzymes to study would be the murine AKR1C enzymes. However, it has been shown that there are no functional orthologs of the AKR1C enzymes in mice (Velica et al., 2009). Among the studies that have been successful, it was found that AKR1B3 (murine aldose reductase) knockout mice exhibited reduced metabolism of advanced glycation end products (AGEs) resulting in AGEs accumulation and atherosclerotic lesion formation (Baba et al., 2009); in addition AKR1B3 knockout mice prevented azoxymethane-induced formation of colonic preneoplastic aberrant crypt foci by a mechanism that may involve reduction of glutathionyl-4-hydroxynonenal to glutathionyl-1,4-dihydroxynonene (Tammali et al., 2009). In another study, AKR1C18 knockout mice which have 20α-hydroxysteroid dehydrogenase were found to have a parturition defect due to the inability to metabolize progesterone (Piekorz et al., 2005).

## Conclusion

Aldo-keto reductases catalyze the metabolic activation of structural diverse PAH *trans*-dihydrodiol proximate carcinogens to yield redox-active and electrophilic PAH *o-*quinones. AKRs also catalyze the two electron reduction of PAH *o-*quinones back to the corresponding cognate PAH catechols, establishing a futile redox cycle which results in ROS formation and subsequent oxidative DNA damage in human lung cells. However, the PAH catechols can be intercepted by COMT, SULTs, and UGTs to form conjugated PAH metabolites, which will terminate the redox-cycling. The toxicological outcome of the *o-*quinone pathway of PAH activation will depend on the balance of the activities of the AKR isoforms and the battery of phase II enzymes implicated in the conjugation process. This balance will be affected by gene expression and polymorphic variants of the enzymes identified.

## Conflict of Interest Statement

The authors declare that the research was conducted in the absence of any commercial or financial relationships that could be construed as a potential conflict of interest.
